# Capacity of middle management in health-care organizations for working with people—the case of Slovenian hospitals

**DOI:** 10.1186/1478-4491-11-18

**Published:** 2013-05-10

**Authors:** Brigita Skela Savič, Andrej Robida

**Affiliations:** 1 Visoka šola za zdravstveno nego Jesenice, College of Nursing Jesenice, Spodnji Plavž 3, 4270 Jesenice, Slovenia; 2 Visoka šola za zdravstveno nego Jesenice, College of Nursing Jesenice, and Prosunt d.o.o., Blejska cesta 13, Zasip 4260, Bled, Slovenia

**Keywords:** Development, Employee, Hospitals, Middle management, Owner responsibility

## Abstract

**Background:**

Effective human resources management plays a vital role in the success of health-care sector reform. Leaders are selected for their clinical expertise and not their management skills, which is often the case at the middle-management level. The purpose of this study was to examine the situation in some fields that involve working with people in health-care organizations at middle-management level.

**Methods:**

The study included eight state-owned hospitals in Slovenia. A cross-sectional study included 119 middle managers and 778 employees. Quota sampling was used for the subgroups. Structured survey questionnaires were administered to leaders and employees, each consisting of 24 statements in four content sets evaluated on a 5-point Likert-type scale. Respondents were also asked about the type and number of training or education programmes they had participated in over the last three years. Descriptive statistics, two-way analysis of variance, Pearson’s correlation coefficient and multiple linear regression were used. The study was conducted from March to December 2008.

**Results:**

Statistically significant differences were established between leaders and employees in all content sets; no significant differences were found when comparing health-care providers and health-administration workers. Employment position was found to be a significant predictor for employee development (*β* = 0.273, *P* < 0.001), the leader–employee relationship (*β* = 0.291, *P* < 0.001) and organizational motivation (*β* = 0.258, *P* < 0.001). Area of work (*β* = 0.113, *P* = 0.010) and employment position (*β* = 0.389, *P* < 0.001) were significant predictors for personal involvement. Level of education correlated negatively with total scores for organizational motivation: respondents with a higher level of education were rated with a lower score (*β* = -0.117, *P* = 0.024). Health-care providers participate in management programmes less frequently than do health-administration workers.

**Conclusion:**

Employee participation in change-implementation processes was low, as was awareness of the importance of employee development. Education of employees in Slovenian hospitals for leadership roles is still not perceived as a necessary investment for improving work processes. Hospitals are state owned and a national strategy should be developed on how to improve leadership and management in Slovenian hospitals.

## Introduction

The ability of a health-care system to provide safe, high-quality, effective, patient-centred services depends on sufficient well-motivated and appropriately skilled personnel operating within service delivery models that optimize their performance [1]. The policies and methods used to manage human resources are at the core of any sustainable solution to health-care system performance and can constrain or facilitate health-care sector reform [2]. Better use of the spectrum of health-care providers and better coordination of patient services through interdisciplinary teamwork have been recommended as part of health-sector reform. Since all health care is ultimately delivered by people, effective human resources management will play a vital role in the success of health-sector reform [3].

Microsystems in health care, as defined by Nelson et al. [4], are small teams working together on a regular basis to serve the needs of a discrete subpopulation of patients. In a microsystem such as a hospital ward, managing the diversity of professional cultures might be the key factor for establishing interdependent ward teams and increasing satisfaction, which leads to improved quality of care [5]. According to Krogstad et al. [5], the only domain of work that significantly predicts high job satisfaction as important for all groups is a positive evaluation of local leadership. Swearingen [6] underscores the ability of leaders to create a positive working environment that motivates and includes employees. The workforce in the health-care sector has specific features that cannot be ignored. For example, motivation can play an integral role in many of the compelling challenges facing health care today [7]. Job satisfaction may be defined as the emotional response to one’s working condition, whereas motivation is the driving force to pursue and satisfy one’s needs. However, job satisfaction and motivation work together to increase job performance and health-care organizations can do many things to increase job satisfaction, primarily by focusing on the motivating interests of the present and future [8].

A systematic literature review conducted by Richardson and Storr [9] identified communication openness, formalization, participation in decision-making and relationship-oriented leadership as key leadership practices. The results of research on leadership styles have led Swearingen [6] to conclude that the way employees perceive their direct leaders plays a major role in the decision of leaders to leave the hospital setting. A study by McCallin and Frankson [10] clearly demonstrated that, in the case of middle management in nursing, leaders are selected for their clinical expertise and not their management skills, which is often the case in health care, especially at the middle-management level. Fernandez et al. [11] established that managers and leaders in health care must assume an active role in conflict mediation and in solving conflicts between different occupational groups in health care, in addition to creating a working environment that fosters efficient interdisciplinary communication.

This article presents some results of the latest study on the capacity of middle management in Slovenian hospitals to work with people on employee development, personal involvement, the leader–employee relationship and organizational motivation. Previous research conducted in Slovenian hospitals has shown that health-care practices have not been managed, implemented or controlled adequately [12], and that they have been founded on a culture and tradition of hierarchical hospital leadership [13]. Conversely, successful change implementation in Slovenian hospitals is mostly the result of teamwork, a form of work not supported or encouraged by the existing hierarchical organizational culture [14,15]. Importantly for the development of middle management, the role and inclusion of middle-level employees has been found to be extremely poor, which represents a great challenge for the top and middle management: they must find a way to tap into the potential of each employee, to meet organizational goals [15]. Quality policy in health-care organizations is not defined clearly enough, making its transfer to lower organizational levels insufficient; this is where leadership and training should play a more significant role [12].

## Materials and methods

The purpose of this study was to assess current practices influencing leader–employee relationships at the level of middle management in participating Slovenian hospitals. The research questions considered how employees are managed, especially in terms of leader–employee relationships, career-development opportunities for employees, inclusion of employees in change implementation and innovation implementation processes, and organizational motivation in participating Slovenian hospitals.

### Study design and measures

A field study of non-experimental design with a descriptive work method was undertaken, using a written, structured questionnaire. The respondents included in the study had to meet two criteria: they must have at least a secondary school diploma, and they must be currently employed at a Slovenian hospital as either a health-care provider or a health-administration worker. Separate questionnaires were used for leaders and employees.

The work of leaders was measured with a 24-item self-report questionnaire consisting of four thematic sets. Similarly, the opinions of employees (both health-care providers and health-administration workers) about their leaders’ work were measured with a 24-item questionnaire divided thematically into four sets. Respondents were asked to rate each item on a Likert-type scale, with responses ranging from 1 (strongly disagree) to 5 (strongly agree).

The questionnaire began with questions about the respondents: age, sex, length of employment in a leading position (for leaders), length of employment in a hospital, level of education, and area of work. At the end of the questionnaire, respondents were also asked questions about the type and number of training or education programmes they had participated in over the previous three years.

The self-report questionnaire was prepared according to a review of relevant literature and previously conducted research. We developed four thematic sets: employee development, personal involvement, leader–employee relationships and organizational motivation. Items on employee development were taken from a previously tested questionnaire used for two studies on health-care management in Slovenia [16,17]. Leader–employee relationships and the contribution of employees towards change were assessed with items designed on the basis of different research [5,18-20]. Opportunities for career development were measured with items previously tested in studies on Slovenian health-care management [16,17,21] and the theoretical background in this area [5,18]. Items used to assess the current situation in organizational management were based on a motivation assessment tool designed by Møller [22]. We did not conduct a pilot study before data collection. The reliability estimates for each thematic set are shown in Table 1.

**Table 1:** Reliability estimates for scales of total set scores

**Leaders together**				**Health-care employees**				**Health-administration employees**			
**Set**	***α***	**Item**	***N***	**Set**	***α***	**Item**	***N***	**Set**	***α***	**Item**	***N***
**1**	0.697	6	117	**1**	0.840	6	615	**1**	0.924	6	112
**2**	0.715	4	117	**2**	0.709	4	643	**2**	0.854	4	117
**3**	0.737	4	117	**3**	0.920	4	635	**3**	0.939	4	119
**4**	0.915	10	115	**4**	0.913	10	613	**4**	0.941	10	117

1, employee development; 2, personal involvement; 3, leader–employee relationship; 4, organizational motivation; *α*, Cronbach’s alpha; *N*, number of respondents who provided answers for all items in a set.

### Sample

The study included eight state-owned hospitals at the secondary and tertiary levels. The participating hospitals were selected with a purposive sample: they actively participated in the Ministry of Health programmes designed to implement and promote quality in health care. Quota sampling was used for subgroups of health-care professionals employed in hospitals. The total number of distributed questionnaires was 1,783, equalling approximately a third of all personnel in the participating hospitals (including medicine, nursing and health administration). The number of returned questionnaires was 897, giving a total response rate of 50.3%. The returned questionnaires represented 17% of all personnel in the participating hospitals.

No sampling was conducted for leaders; the questionnaires were distributed to all leaders at the hospital ward, unit and department levels. Of the 190 questionnaires distributed to health-care leaders, 105 were returned, giving a response rate of 55%, which was close to that of health-administration leaders, with 27 distributed questionnaires and 14 returned questionnaires (a response rate of 52%).

For health-care employees, the number of questionnaires distributed was 1,357 and the response rate was 48%, with 657 returned questionnaires. A further 209 questionnaires were distributed among health-administration employees and 121 were returned, giving a response rate of 58%.

The respondents were predominately female (706 respondents, or 80% of the sample, were female). The total share of female respondents is higher in the leaders group than in the employees group. The distribution for the level of education shows that the majority of respondents (46%) have a secondary school diploma, followed by a degree from a professional college (25.5%) and a university degree (12.1%). The majority of leader respondents are either nursing professionals (*N* = 42) or medical professionals (*N* = 37). The total number of nursing employees was 465, followed by 77 health-administration employees and 63 medical employees.

### Data collection

We first obtained permission to conduct the study from hospital directors, who took responsibility for ethics permission for each institution according to that institution’s regulations (the Professional Council of each hospital is responsible for studies on health-care workers). Each hospital employed research coordinators who were professionals from the first or second leadership levels, whose assignments included implementing quality standards in hospitals and conducting nursing leadership tasks. With the assistance of hospital management and research coordinators, the questionnaires were distributed on a selected day to all employees in units who were present on that day. The respondents were given seven days to fill out the questionnaires and leave them in a folder in their unit, a process that ensured the respondents’ anonymity. The respondents were informed of their rights as participants in the study by an introductory letter (stating the voluntary nature of the study, anonymity and confidentiality), which was part of the questionnaire. They placed the questionnaires in envelopes prior to returning them at the predetermined collection place.

The study was conducted from March to June 2008. We did not need the approval of an ethics committee. The research was supported by the Ministry of Health of the Republic of Slovenia (contract no. C2711-07Y000217).

### Data analysis

Frequency divisions were performed for all the items researched, and appropriate descriptive statistics were calculated. Total results for individual sets of items were computed, with the exception of the first set (respondents’ profile) and the last set (information on training and education programmes). Internal consistency for the total set results was assessed using Cronbach’s alpha. Next, the differences between total scores were calculated before conducting further analysis. To do this, we used one-way analysis of variance for the age and length of employment variables, the Kruskal-Wallis non-parametric test for the number of subordinates or employees, the Mann–Whitney U test for the number of years at a leading position, and Fisher’s exact test for sex, level of education, and area of work. A two-way analysis of variance was then performed for each total score, with the two intergroup factors being the type of work performed (health-care provision or health administration) and respondent’s position (leader or employee). The model residual normality assumption for these analyses was examined with a P–P plot, which did not reveal significant differences for any of the total scores. Because the interaction effect was not statistically significant for any of the models, we analysed the correlation between general characteristics and total scores for all respondents together.

Pearson’s correlation coefficient was used to analyse the correlation of total set scores, and multiple linear regression was used to assess the correlation between respondents’ characteristics and total set scores. Prior to that, we made sure for each of the total set scores that the level of education could be expressed as a variable number by applying the one-way analysis of variance (or, in the case of non-homogeneous variance demonstrated by Levene’s test, we applied Welch’s test for unequal variances) and post-hoc comparisons (with Scheffe’s post-hoc test or the Games–Howell test). The level of statistical significance was set at *P* < 0.05.

## Results

The item results for given sets, showing leaders and employees separately, and a two-way analysis of variance for total scores are shown in Table 2. No significant differences were found to exist in the total mean scores for any of the sets when comparing health-care providers and health-administration workers. However, statistically significant differences were established between leaders and employees in all sets. The two-way analyses of variance results for total set scores are shown in Table 3.

**Table 2:** Item results for given sets, separately for leaders and employees and a two-way analysis of variance for total scores

**Questionnaire items: leaders or employees**	**Leaders**						**Employees**					
	**Health-care provision**			**Health administration**			**Health-care provision**			**Health administration**		
**Set 1: employee development**	Two-way analysis of variance for total scores: position (leader : employee) *P *< 0.001; area (health care : administration) *P *= 0.435											
	*N*	*M*	SD	*N*	*M*	SD	*N*	*M*	SD	*N*	*M*	SD
Each employee at the unit I lead is familiar with their hospital training and development programme for the next four years.	104	3.34	0.84	14	3.43	1.09	645	2.71	1.11	118	2.85	1.22
*I am familiar with my hospital training and development programme for the next four years.*												
When preparing the training and development programme for each employee, I always include their views and needs.	103	4.02	0.73	14	3.86	0.86	644	2.64	1.12	115	2.75	1.32
*I participate in the preparation of my annual training and development programme and am given the opportunity to present my views and needs.*												
I hold a meeting at least once a year with each employee in my unit to discuss their work and involvement in the unit.	103	3.90	0.91	14	3.86	1.10	643	3.52	1.22	118	3.53	1.27
*I meet my leader at least once a year to discuss my work and my involvement in the unit.*												
I equally promote the development of an area of work at the unit I lead and the development of employees at my unit.	103	4.40	0.65	14	4.21	0.70	642	3.71	1.08	120	3.37	1.22
*The leader of the unit where I work promotes the development of an area of work at that unit and the development of its employees equally.*												
As a unit leader I encourage the capacity for innovation, learning new skills and applying them in practice in my employees.	103	4.55	0.61	14	4.43	0.76	648	3.84	1.02	119	3.49	1.21
*The leader of the unit where I work encourages the capacity for innovation, learning new skills and applying them in practice.*												
I prepare the annual training and education programme together with the employees at my unit and make sure it is congruent with unit and hospital needs . *The leader of the unit where I work prepares the annual training and education programme together with their employees and makes sure it is congruent with unit and hospital needs.*	103	4.23	0.76	14	4.07	1.07	647	3.37	1.17	118	3.05	1.35
**Set 2: personal involvement**	Two-way analysis of variance for total scores: position (leader: employee) *P *< 0.001; area (health care: administration) *P *= 0.352.											
I give employees an opportunity to suggest improvements for key projects being introduced into practice.	104	4,45	0.64	14	4.29	0.73	648	2.79	1.06	119	2.95	1.25
*My work at the hospital provides me with an opportunity to suggest improvements for key projects being introduced into practice.*												
I create conditions for employees to contribute their knowledge in change-implementation processes.	104	4.41	0.60	14	4.50	0.52	649	3.05	0.99	118	3.12	1.13
*I can contribute my knowledge in the change-implementation processes affecting my area of work through well managed work process improvement groups.*												
I give employees an opportunity to be actively involved in the change-implementation processes at the hospital.	104	4.23	0.69	14	4.43	0.65	646	3.64	0.93	118	3.79	1.06
*I desire to be actively involved in the change-implementation processes at the hospital.*												
I am satisfied with my status and leadership role in the hospital.	104	3.85	0.95	14	4.21	0.70	648	2.96	1.08	119	3.03	1.33
*I am happy with my status and role in our hospital. My suggestions and wishes for my professional development are taken into account.*												
**Set 3: leader–employee relationship**	Two-way analysis of variance for total scores: position (leader : employee) *P *< 0.001; area (health care : administration) *P *= 0.371											
I offer my employees support and try to understand them when they turn to me with problems.	103	4.50	0.62	14	4.29	0.83	646	3.73	1.06	121	3.62	1.13
*The leader of the unit where I work communicates with employees by showing their support for work-related problems.*												
I provide employees with clear feedback on their work.	104	4.30	0.75	14	4.29	0.47	646	3.73	1.07	120	3.63	1.17
*My leader provides me with clear feedback on my work*.												
When expressing dissatisfaction with an employee’s work, I encourage them to improve instead of displaying anger.	104	4.09	0.70	14	3.93	0.73	645	3.67	1.03	119	3.61	1.15
*When my leader expresses dissatisfaction with my work or results, they encourage me to improve instead of displaying anger*.												
I listen to my colleagues attentively and am open to their suggestions, even if I don’t agree with their proposals.	105	4.32	0.64	14	4.14	0.66	642	3.72	1.00	120	3.64	1.12
*The unit leader is open to suggestions from employees, even if they don’t agree with the proposals*.												
**Set 4: motivation**	Two-way analysis of variance for total scores: position (leader : employee) *P* < 0.001; area (health care : administration) *P* = 0.228											
The goals of the organization are clear; all employees are familiar with them.	105	3.71	0.99	14	3.86	0.86	647	3.11	1.09	121	3.31	1.18
The activities of all employees are in synergy; we all work towards the same goal, our actions are not conflicting.	104	3.63	0.84	14	3.93	0.73	645	3.01	1.03	119	3.06	1.07
Overall, we are successful in meeting our goals: our actions are goal-driven and not controlled by emergency calls, cell phones, e-mails or urgent tasks coming from our superiors.	105	3.69	0.78	14	3.93	0.62	635	3.28	0.94	119	3.26	1.04
We all try to do the best in everything we do.	105	4.17	0.75	14	4.07	0.83	649	4.02	0.97	120	3.84	1.05
There is a strong sense of optimism present in the organization.	105	3.37	0.92	14	3.71	1.20	644	3.13	1.09	120	3.13	1.19
We do not give up when things become difficult or go wrong.	105	3.84	0.84	14	3.86	0.66	648	3.62	0.97	120	3.60	1.06
People in our organization truly enjoy their work; their energy levels are very high.	104	3.36	0.91	14	3.50	0.94	641	2.80	1.09	120	2.95	1.21
People in our organization are self-motivated; we do not require external incentive to perform our work.	105	3.30	0.91	14	3.43	0.94	641	3.00	1.04	119	2.98	1.17
Our organization is characterized by individual commitment, a sense of responsibility, loyalty and taking initiative.	103	3.55	0.89	14	3.79	0.80	641	3.23	0.95	120	3.18	1.12
There is an overall sense of satisfaction with the organization and working conditions among the employees.	105	3.21	0.88	14	3.64	0.84	643	2.74	1.07	119	2.94	1.24

*N*, number of respondents who answered an item in the set; *M*, mean value on a 1 to 5 scale; SD, standard deviation.

**Table 3:** Two-way analysis of variance results for total set scores

***P***	**Sets**			
**Effect**	**Employee development**	**Personal involvement**	**Leader–employee relationship**	**Organizational motivation**
**Area of employment**	0.435	0.352	0.371	0.338
**Employment position**	<0.001	<0.001	<0.001	<0.001
**Interaction**	0.827	0.925	0.849	0.512

### **Set 1**: **employee development**

Overall, leaders rated their active participation in employee development extremely highly, with the most frequent response to the first three items in Table 2 on employee participation in career development being “mostly agree” and for the items describing the leader’s motivational role in employee development being “completely agree”. The lowest-ranked item for both leaders in health-care provision (mean = 3.34, SD = 0.84) and health administration (mean = 3.43, SD = 1.09) was, “Each employee at the unit I lead is familiar with their hospital training and development programme for the next four years.”

Conversely, employees rated their leaders significantly lower on the issue of implementing employee-development activities. The differences were significant only for total mean scores (*P* < 0.001), but not between health-care provision and health-administration groups (*P* = 0.435). Employees in health-care provision (mean = 2.71, SD = 1.11) and health administration (mean = 2.85, SD = 1.22) are not very familiar with their training and development programmes. The rate of participation in preparing the programme (where employees have the opportunity to present their views and needs) was low for health-care providing employees (mean = 2.64, SD = 1.12), and for health-administration employees (mean = 2.75, SD = 1.32). Employees were found to agree slightly with the items on employee participation in the organization of their unit (mean health care (MHC) = 3.52, SD = 1.22; mean health administration (MHA) = 3.53, SD = 1.27) and on employee participation in the preparation of the annual training and education programme (MHC = 3.37, SD = 1.17, MHA = 3.05, SD = 1.37). Employees mostly agreed with the item about the importance of leaders in promoting capacity for innovation and learning new skills (MHC = 3.84, SD = 1.02; MHA = 3.49, SD = 1.21). Health-care providing employees expressed a higher rate of agreement for the need for equal development of an area of expertise and the development of employees working in that area than did health-administration employees (MHC = 3.71, SD = 1.08; MHA = 3.37, SD = 1.22).

### Set 2: personal involvement

A significant difference between leader and employee opinions in mean total scores (*P* < 0.001) for employee involvement and consideration of their suggestions, skills and capabilities was established, whereas no significant differences were found to exist between health-care providers and health-administration workers (*P* = 0.352). Employee involvement processes are not encouraged sufficiently for either health-care providers (mean = 2.79, SD = 1.06) or health-administration employees (mean = 2.95, SD = 1.25); however, the leaders’ opinion on successful implementation of these programmes is significantly higher. A similar trend was seen in the previous set of items on employee development. Employees perceive a lack of their involvement in change-implementation processes and work improvement strategies, which means that their potential remains insufficiently tapped (MHC = 3.05, SD = 0.99; MHA = 3.12, SD = 1.13). We found a surprisingly low desire among employees to be actively involved in change-implementation processes (MHC = 3.64, SD = 0.93; MHA = 3.79, SD = 1.06), although leaders expressed a conviction that their employees were given the opportunity to be actively involved (MHC = 4.23, SD = 0.69; MHA = 4.43, SD = 0.65). Overall, leaders were satisfied with their status and role in the hospital (MHC = 3.85, SD = 0.95; MHA = 4.21, SD = 0.70), unlike employees, whose satisfaction level was found to be much lower (MHC = 2.96, SD = 1.08; MHA = 3.03, SD = 1.33).

### Set 3: leader–employee relationship

In leaders, the self-reported rate for their active role in offering employees help with problems, providing feedback, encouraging improvements and expressing openness for new ideas was high. Both health-care and health-administration employees perceive this role of leaders as significantly lower (*P* < 0.001), but their responses still indicate a prevalent agreement with leaders’ ability to establish good rapport with employees (MHC = 3.73, SD = 1.06; MHA = 3.62, SD = 1.13), to provide feedback (MHC = 3.73, SD = 1.07; MHA = 3.63, SD = 1.17), to encourage improvements in the work process (MHC = 3.67, SD = 1.03; MHA = 3.61, SD = 1.15), and to accept different ideas (MHC = 3.72, SD = 1.00; MHA = 3.64, SD = 1.12).

### Set 4: organizational motivation

The items used to assess organizational motivation were the same for leaders and employees. Overall, leaders rated organizational motivation higher than employees (*P <* 0.001), even though the differences for this set of items were smaller than in the other sets. There were no significant differences between health-care providers and health-administration workers (*P* = 0.228). The goals of the organization appear to be less clear to the employees than to the leaders, and the same is true for perceived synergy in the organization. Both leaders and employees mostly agree that their intention is to conduct the best possible work, and slightly agree that optimism is felt in the organization.

Employees expressed a significantly lower rate of agreement (MHC = 3.11, SD = 1.09; MHA = 3.31, SD = 1.18) than their leaders on the clarity of organizational goals (MHC = 3.71, SD = 0.99; MHA = 3.86, SD = 0.86). Employees tend to rate the perceived synergy in the organization (MHC = 3.01, SD = 1.03; MHA = 3.06, SD = 1.07) lower than do their leaders. Employees and leaders mostly agree that they try to do their best, and slightly agree that a strong sense of optimism is present in the organization and that despair does not help solve problems in an organization. Slight agreement was expressed with the idea that individuals working in the organization do not require external incentive to perform their work, and that the organizational advantages include individual commitment, a sense of responsibility, loyalty and taking initiative.

Correlation analysis for total set scores revealed a significant positive correlation between all sets at the level *P* < 0.001, both for leaders and employees (Table 4).

**Table 4:** Correlations for total set scores

		**Personal involvement**	**Leader–employee relationship**	**Organizational motivation**
**Leaders**	Employee development	0.451**	0.408**	0.401**
	Personal involvement		0.489**	0.498**
	Leader–employee relationship			0.364**
**Health-care provision employees**	Employee development	0.617**	0.650**	0.438**
	Personal involvement		0.508**	0.450**
	Leader–employee relationship			0.484**
**Health-administration employees**	Employee development	0.787**	0.708**	0.654**
	Personal involvement		0.664**	0.671**
	Leader–employee relationship			0.575**

(Pearson’s *r*; ** *P *< 0.001; * *P *< 0.05).

The correlation between total set scores and respondent characteristics is shown in Table 5. Employment position was identified as the strongest predictor for all sets (the expected results were a grade higher for leaders than for employees with the same characteristics). Level of education was a significant positive predictor for Set 2 (personal involvement) and a negative predictor for Set 4 (motivation). Finally, area of work was found to correlate significantly with total set scores for Set 3 (personal involvement), where other respondent characteristics were the same.

**Table 5:** Multiple linear regression results for predicting total set scored based on respondent characteristics

**Set**	**Characteristic**	***b***	**SE**_***b***_	***β***	***P***
**1** (*R*^2^ = 0.09)	Area of employment (health-administration or health-care providers)	-10.747	20.446	-0.034	0.475
	Employment position (leader vs employee)	130.447	20.124	0.273	<0.001
	Age (years)	0.007	0.147	0.004	0.965
	Sex (F/M)	10.941	10.750	0.045	0.268
	Length of employment (years)	-0.047	0.141	-0.027	0.740
	Number of subordinate employees (leaders) or number of employees (employees)	0.004	0.018	0.007	0.845
	Level of education (secondary … PhD)	10.034	0.675	0.077	0.126
	Area of work = medical professional (yes/ no)	0.235	20.604	0.004	0.928
	Area of work = nursing professional (yes/no)	-0.009	20.010	0.000	0.996
	Training and education (yes/no)	0.676	10.445	0.019	0.640
**2** (*R*^2^ = 0.21)	Area of employment (health-administration or health-care providers)	50.526	20.149	0.113	0.010
	Employment position (leader vs employee)	180.069	10.867	0.389	<0.001
	Age (years)	-0.018	0.130	-0.011	0.890
	Sex (F/M)	-0.589	10.538	-0.014	0.702
	Length of employment (years)	0.039	0.124	0.024	0.752
	Number of subordinate employees (leaders) or number of employees (employees)	0.021	0.016	0.045	0.198
	Level of education (secondary … PhD)	10.652	0.593	0.130	0.006
	Area of work = medical professional (yes/ no)	-0.166	20.288	-0.003	0.942
	Area of work = nursing professional (yes/no)	20.546	10.766	0.075	0.150
	Training and education (yes/no)	20.019	10.272	0.060	0.113
**3** (*R*^2^ = 0.06)	Area of employment (health-administration or health-care providers)	10.040	20.535	0.020	0.682
	Employment position (leader vs employee)	140.441	20.192	0.291	<0.001
	Age (years)	-0.187	0.153	-0.102	0.223
	Sex (F/M)	20.855	10.812	0.065	0.116
	Length of employment (years)	-0.047	0.146	-0.027	0.746
	Number of subordinate employees (leaders) or number of employees (employees)	0.010	0.019	0.021	0.577
	Level of education (secondary … PhD)	-0.155	0.700	-0.011	0.824
	Area of work = medical professional (yes/ no)	10.017	20.700	0.019	0.706
	Area of work = nursing professional (yes/no)	0.257	20.092	0.007	0.902
	Training and education (yes/no)	-10.071	10.499	-0.030	0.475
**4** (*R*^2^ = 0.05)	Area of employment (health-administration or health-care providers)	30.719	20.269	0.080	0.102
	Employment position (leader vs employee)	110.409	10.957	0.258	<0.001
	Age (years)	0.129	0.136	0.079	0.343
	Sex (F/M)	-10.140	10.615	-0.029	0.481
	Length of employment (years)	-0.196	0.130	-0.124	0.133
	Number of subordinate employees (leaders) or number of employees (employees)	0.016	0.017	0.036	0.347
	Level of education (secondary … PhD)	-10.413	0.624	-0.117	0.024
	Area of work = medical professional (yes/ no)	0.675	20.407	0.014	0.779
	Area of work = nursing professional (yes/no)	30.526	10.872	0.108	0.060
	Training and education (yes/no)	-20.285	10.336	-0.071	0.088

1, Employee development; 2, Personal involvement; 3, Leader–employee relationship; 4, Organizational motivation; *b*, Regression coefficient; SE_*b*_, standard regression coefficient error, *β*, standard regression coefficient.

Table 5 shows that leaders rated employee development (Set 1) significantly higher than employees (*P* < 0.001). The same was also true for the leader–employee relationship (Set 3; *P* < 0.001) and personal involvement (Set 2). Higher scores for personal involvement were identified for respondents with higher education (*P* = 0.006) and respondents working as health-care providers (*P* = 0.010).

In terms of respondent characteristics, employment position was found to be a significant predictor for employee development (Set 1), with leaders averaging higher scores than employees (*β* = 0.273, *P* < 0.001). Area of work (*β* = 0.113, *P* = 0.010) and employment position (*β* = 0.389, *P* < 0.001) were significant predictors for personal involvement (Set 2). For the leader–employee relationship (Set 3), employment position was found to be a significant predictor (*β* = 0.291, *P* < 0.001). Employment position was also a significant predictor for motivation (Set 4), as was level of education. Results have shown that leaders rate Set 4 higher than employees (*β* = 0.258, *P* < 0.001), and that the level of education correlates negatively with total scores for Set 4: respondents with a higher level of education rated Set 4 with a lower score (*β* = -0.117, *P* = 0.024) (Table 5).

The correlation between respondent characteristics and their participation in at least one training and education programme over the previous three years is shown in Table 6. Correlation analysis showed the following respondent characteristics to be statistically important: leader, male, higher education, health-care provider, and older.

**Table 6:** Correlation between respondent characteristics and participation in at least one training and education programme

**Variable**	**Description**	**Training and education programme**		***P***
		**No**	**Yes**	
Area of employment	Health-care provision	448	314	0.506
		58.8%	41.2%	
	Health administration	84	51	
		62.2%	37.8%	
Employment position	Leader	26	93	<0.001
		21.8%	78.2%	
	Employee	506	272	
		65.0%	35.0%	
Sex	Male	82	94	<0.001
		46.6%	53.4%	
	Female	443	263	
		62.7%	37.3%	
Level of education	Secondary	299	86	<0.001
		77.7%	22.3%	
	Junior/professional college	162	155	
		51.1%	48.9%	
	University	20	81	
		19.8%	80.2%	
	MSc or PhD	4	30	
		11.8%	88.2%	
Area of work	Medical professional	33	70	<0.001
		32.0%	68.0%	
	Nursing professional	333	176	
		65.4%	34.6%	
	Health administration	107	109	
		49.5%	50.5%	
Age		mean = 38.3	mean = 40.0	0.016
		(SD = 10.1)	(SD = 9.8)	
Length of employment		mean = 15.3	mean = 15.0	0.692
		(SD = 10.7)	(SD = 10.1)	
Number of subordinate employees (leader) or of employees (employee)		mean = 28.0	mean = 24.6	0.185
		(SD = 41.8)	(SD = 24.3)	
Number of years in leading position		mean = 7.8	mean = 8.7	0.593
		(SD = 5.8)	(SD = 7.1)	

Descriptive variables: frequency, category shares and Fisher’s exact test statistics; numeric variables: mean, standard deviation and Student’s *t*-test statistics; SD, standard deviation.

Multiple logistic regression yielded two significant independent predictors for participation in training and education programmes: employment position (*P* = 0.003), where leaders scored higher than employees, and level of education (*P* < 0.001), where higher education means a greater likelihood for participation in a training and education programme. Borderline statistical significance was established, with men having an advantage over women in the frequency of participation in training and education programmes (*P* = 0.057). The differences for area of work and age yielded by univariate comparisons are primarily the result of interrelation between respondent characteristics (e.g., a greater share of older workers among leaders and a higher level of education among medical professionals).

Table 7 shows the differences between respondent groups according to type of subject covered in training and educational programmes, and Table 8 shows differences for level of education. Regardless of the training and education subject, leaders were found to participate in these programmes significantly more than employees; moreover, health-care providers participated in Programmes 4, 5, 6, 7 and 11 less frequently than health-administration workers. As expected, training and education programmes that are not part of a full-time study programme are a more common alternative. In postgraduate studies, leaders were shown to have a significant advantage over employees, and health-administration workers had a significant advantage over health-care providers. Conversely, postgraduate-level training and education programmes organized by and in the hospital are more frequently attended by leaders than employees, and by health-care providers than health-administration workers; in postgraduate-level programmes organized outside the hospital, leaders were shown to have an advantage over employees.

**Table 7:** Differences in respondent groups according to subject of education and training programmes

**Subject of programme**		**Group**				**Total**	***P***	
		**HCL**	**HAL**	**HCE**	**HAE**		**Position**	**Area of employment**
1	Professional issues	76.2%	57.1%	32.3%	30.6%	37.6%	<0.001	0.411
2	Quality in health care	61.0%	35.7%	15.2%	17.4%	21.2%	<0.001	0.817
3	General issues	48.6%	21.4%	14.9%	20.7%	19.7%	<0.001	0.538
4	Health-care system functioning	23.8%	35.7%	4.3%	13.2%	8.2%	<0.001	<0.001
5	Creating a vision, setting goals, etc.	37.1%	35.7%	7.5%	18.2%	12.8%	<0.001	0.002
6	Leadership of employees	46.7%	21.4%	5.6%	15.7%	12.0%	<0.001	0.024
7	Development of employees	23.8%	14.3%	2.7%	10.7%	6.5%	<0.001	0.006
8	Change implementation	37.1%	28.6%	6.4%	10.7%	10.9%	<0.001	0.275
9	Process organization	38.1%	28.6%	6.7%	11.6%	11.4%	<0.001	0.229
10	Total quality management	38.1%	21.4%	7.8%	11.6%	12.0%	<0.001	0.575
11	Health-care financing, etc.	29.5%	21.4%	2.1%	11.6%	6.9%	<0.001	0.001

HAE, health-administration employees; HAL, health-administration leaders; HCE, health-care employees; HCL, health-care leaders; *P* values obtained from logistic regression model.

**Table 8:** Differences in respondent groups according to type of training and education programmes

**Type of training and education**	**Groups**				**Total**	***P***	
	**HCL**	**HAL**	**HCE**	**HAE**		**Position**	**Area of employment**
**Undergraduate studies**	6.7**%**	7.1%	11.4%	13.2**%**	11.0%	0. 116	0.572
**Postgraduate studies**	15.2%	21.4%	3.7%	12.4%	6.5%	<0.001	<0.001
**Postgraduate programmes within the hospital**	60.0%	7.1%	19.3%	13.2%	23.1%	<0.001	0.004
**Postgraduate programmes outside the hospital, seminars**	72.4%	71.4%	18.6%	18.2%	25.6%	<0.001	0.903

HAE, health-administration employees; HAL, health-administration leaders; HCE, health-care employees; HCL, health-care leaders; *P* values obtained from logistic regression model.

## Discussion

Overall, the results show no significant difference between the respondents’ occupational groups in their agreement with the questionnaire statements. This is contrary to previous research findings for Slovenian hospitals [12-15,23-25], where statistically significant differences were established between occupational groups for comparable variables. Nevertheless, the results in this study have shown significant differences in the rate of agreement between leaders and employees, in agreement with previous research results.

Leaders were found to rate their active participation in employee development extremely highly, while the ratings of employees were significantly lower regarding the role of leaders in this field. Moreover, employee responses revealed that they were not familiar with the training and development programmes for the next four years, and not sufficiently included in them. The participating hospitals moderately encourage the culture of annual interviews, and moderately promote professional development, the acquisition of knowledge and skills, and their practical application. The needs for education and training required to facilitate work at a department are partially reflected in employees’ education needs. The results suggest that leaders are critical of the principal human resource management activity—personal training and development programmes—since they responded to this item with the lowest mean value. In general, human resource management in the participating hospitals is underdeveloped. Interestingly, other studies on human resource management “posit, although rarely directly test, that the hospital-level outcomes increase as a function of human resource management systems increasing employee participation behaviour [26].” It should be remembered, however, that the outcomes of clinical interventions in Slovenian health care are not yet systematically monitored, making it difficult to assess the impact of employee development on the quality of work.

Managers at the participating hospitals were reluctant to include employees in the decision-making and change-implementation processes. Moreover, the inclusion of employees in their development and the complex operations of an organization were low. According to Plsek [27], a health-care organization is a complex system that prioritizes interpersonal relations, structures, processes and models, the personalities of individuals, a high degree of flexibility, experimentation and searches for optimum solutions, the impact of non-linear change and the inclusion of the organization into the broader system. In their efforts to implement development and innovation strategies, health-care organizations often face the unconscious remains of Taylorism from the era of industrial management on the one hand, and constant disagreement between managers and doctors or between groups of health-care professionals on the other. This fact has been demonstrated by previous research results in Slovenian health care, especially for doctor–nurse relations [13,15,28]. Differences in opinion towards integrated patient care between doctors and nurses, and hierarchical doctor–nurse relationships have led to the formation of strong subcultures of doctors and nurses [29], as has been demonstrated by previous research [13-15,21,28] in Slovenia.

Surprisingly, the desire of employees to become actively involved in change implementation at their hospitals was low, even though leaders believe that employees have the opportunity for active involvement, a fact highlighting employee passivity. The passive role of employees in Slovenian hospitals was previously shown by Skela Savič et al. [14], establishing that hierarchy in Slovenian hospitals has been accepted and internalized by the employees and that employees do not desire changes in the organizational culture. Consequently, the readiness of employees to accept responsibility for change could be questioned. Kane-Urrabazo [30] has suggested that managers must put support systems and other mechanisms into place that encourage employees to empower themselves and to flourish, thus increasing their own effectiveness as well as that of the organization.

Leaders were predominately satisfied with their status and leadership roles in the hospital; employees’ satisfaction levels were much lower. Therefore, leaders should work towards the delegation of authority and recognition of personnel efforts; opportunities for promotion and job enrichment must be a part of hospitals’ human resources strategy [31,32].

Areas where leaders could improve their performance include work-related problem solving and providing clear feedback on employees’ work. Participating employees were found to be insufficiently included in working teams, and their intellectual capital and experience were not fully tapped. In addition, employees’ satisfaction with their status and role in the hospital was low. Employees feel that their suggestions and wishes for professional development should be taken into account more; they also wish to be actively involved in the change-implementation process at the hospital. These results are congruous with those of Skela Savič and Pagon [24]. Generally speaking, we can conclude that participating employees are only partially satisfied with their involvement, status and roles in the hospitals where they work. Indirectly, these results can be explained by previous research results on organizational culture in Slovenian hospitals, which was found to be directed predominately towards hierarchy, control and supervision [14,15,21]. Several international studies have demonstrated a positive correlation between organizational culture and employee satisfaction or the efficiency of an organization [33-37]. This is an important reason for managers and leaders to be aware of their contribution in establishing organizational culture. Managers and leaders in health care who create and manage organizational culture, build teamwork and lead the personal development of each employee must accept responsibility for the results of existing personal involvement and start to work on interprofessional collaboration within the organization and outside of it [14]. Establishing good working teams within the micro-unit is a vital challenge for local leaders; managing the cultural diversity of professions is a central part of that challenge [5,38].

Overall, leaders rated organizational motivation higher than did their employees, but the differences between the two groups for this set were not as pronounced as for the other sets. Organizational goals tend to be less clear to the employees than to the leaders, and the same holds true for the perceived synergy in an organization. Leaders and employees mostly agree that their intention is to perform the best possible work and slightly agree that optimism is present in the organization. A significant difference was established for satisfaction with working conditions, where employees were significantly less satisfied than leaders. A slight agreement was expressed with the idea that individuals working in the organization do not require external incentive to perform their work, and that the organization’s advantages are individual commitment, a sense of responsibility, loyalty and taking initiative. Lambrou et al. [39] have recognized that health-care professionals tend to be motivated more by intrinsic factors (e.g. meaningfulness of work, strong interpersonal relationships, respect), implying that this should be a target for effective employee motivation.

These results confirm the hypothesis that leaders should consider different aspects of job satisfaction for different employees, because job satisfaction predictors vary according to individuals. Krogstad et al. [5] found the most common predictors of job satisfaction to be good leadership, professional development, good communication and support from the immediate superior. Low motivation leads to the insufficient transfer of knowledge, the underutilization of available resources, and weak health-care system performance [40,41]. Organizational motivation levels can be attributed directly to the actions of respondent leaders [42]. Leaders should be inspiring and motivate employees to work better [43]; they should have a clear self-image based primarily on successful achievement of past goals; they should be decisive and committed to their work [44]; and they should use the transformational leadership style to significantly increase employee motivation [43,45].

Multiple linear regression analyses revealed that employment position was the crucial predictor for the four examined variables, all of which can be significantly explained by leaders and their actions, a fact that underscores the importance of leadership in hospitals. Similar results were obtained by Krogstad et al. [5]. For personal involvement, a positive correlation was established for the explanatory variables “area of employment” (health-care providers or health administration) and “level of education”, which means that a higher level of education increased the level of agreement with the statements on leader–employee relationship. By contrast, level of education correlated negatively with motivation: respondents with a lower level of education rated motivation lower.

The predictive power of leadership was demonstrated for all variables. A previous study by Skela Savič et al. [14] demonstrated that teamwork was the key predictor of personal involvement in change-implementation processes, making leaders’ education, training and career development crucial [10]. In fact, Sellgren et al. [46] established that employees desire a leader with a clearer leadership style than the leader personally deems appropriate, that employees desire leaders to adopt a more active leadership role, and to be clearer in work-related instructions. These factors should be considered when selecting health-care leaders, and when planning their continuous professional development.

In interpreting the results of this research, it is important to note that leaders in Slovenian health care are primarily selected for their professional merit and often have little experience in leadership, which means that leadership styles are frequently outdated, as has been demonstrated by previous research [16,17,24]. When implementing improvements in Slovenian health-care system leadership, it should not be forgotten that researchers [47,48] have pointed to a close correlation between the quality of treatment and leadership in health care. Employees will not support change-implementation processes in their working environment unless they play an active role in these processes. This emphasizes the role of leaders, who should include individuals in change-implementation processes, because that is an important predictor for successful functioning of a health-care system. These results have shown low levels of employee participation in change-implementation processes, resulting in moderate employee satisfaction with status and role at the hospital.

These results clearly demonstrate that Slovenian hospitals lack a comprehensive career-development system—the percentage of leaders who have participated in postgraduate education programmes is low, and the most frequently attended training and education programmes were those from their own professional fields. A higher participation of health-care leaders in quality-implementation programmes over the last seven years is the result of a systematic programme launched by the Ministry of Health for comprehensive quality implementation in Slovenian hospitals, under which the Ministry and other health-care associations organized training and education programmes. The results for participation of leaders at other training and education programmes relevant for middle management reveal a participation rate of less than 40%, indicating a non-systematic nature for achieving knowledge goals in those areas relevant for quality middle-management work (vision, strategy, employee leadership, change implementation, organization of work processes, comprehensive quality management, health-care funding, etc.) Education is an important factor for organizational development. Research conducted by Skela Savič et al. [23] in nine Slovenian hospitals has shown that leadership performance in participating hospitals correlated with the type and extent of previous training and education programmes for leaders and employees. In hospitals where only leaders participated in training and education programmes, employees tended to rate their leadership with lower mean scores than they did in other hospitals, emphasizing the fact that employees, too, require the knowledge and skills to understand their leaders’ instructions, to participate in teamwork, and to understand the change-implementation process. These results showed that leaders received significantly more training and education than employees, and that health-administration leaders received significantly more education and training in health-care system management, leadership, employee development and health-care funding than did health-care leaders. Education of employees in Slovenian hospitals is still not perceived as a necessary investment for improving work processes, and the education of leading health-care workers in management skills is still not high enough.

This study’s results demonstrate a clear gap between leaders and employees. When interpreting the results, we need to keep in mind that almost all Slovenian hospitals are state owned and that the state is, in fact, responsible for their administration by appointing directors. Moreover, state representatives are members of supervisory boards of each state-owned hospital, which undoubtedly plays a major role in the management of hospitals and has an important effect on leader–employee relationships. Another important issue is that health-care employees are part of the public sector, whose salaries include payments from the Health Insurance Institute of Slovenia and voluntary health insurance companies for services provided. The “public-servant” status results in an extremely rigid payment system with limited room for manoeuvre concerning personnel motivation. Additionally, the “public-servant” status allows limited opportunities for changes in the hierarchical structure of a hospital. In terms of health-care policy, it is extremely important that the state, as the owner of hospitals, take responsibility for the existing situation, described previously in other Slovenian studies [12-17,21,23-25,28].

As with any survey, the issue of representative sampling presents itself. The questionnaire was distributed to all leaders of participating hospitals, who were selected with a purposive sample. The employee sample was cross-sectional, purposive, and used quota sampling. Possibly more concerning is the response rate, and, speculatively, the opinions of those who chose not to participate in the research [49-51]. We believe, however, that the response rate data have been appropriately presented [52]. Although response rates in participating hospitals differed widely, the purpose of this research was not interhospital comparison but situation assessment for all hospitals. Future in-depth research is required on the impact of middle management for the performance and efficiency of health-care organizations and the health-care system as a whole. The data were collected at the start of the global recession in Slovenia. We suggest that new studies be conducted in the future to measure the impact of austerity measures in 2012 and 2013 on the performance and efficiency of middle management on health-care organizations in Slovenia.

## Conclusion

This research has confirmed some previous results on this topic for health-care systems in Slovenia and around the developed world. More importantly, the results also represent new findings in the examined areas of middle management.

The middle-management system of values in the participating hospitals was found to overestimate professional knowledge and skills and show a lack of emphasis on management skills. Moreover, the career-development plan for hospital leaders, specifically education programmes and lifelong learning programmes, was inefficient—the percentage of leaders attending postgraduate programmes was low, while the majority of attended training courses and education programmes dealt with the specific field of expertise for each leader. There was little employee participation in change-implementation processes, or awareness of the importance of employee development. When discussing the obtained results, we should not forget that the research only included hospitals with high change-implementation goals, so the research should be conducted again in all Slovenian hospitals to establish the possible differences in middle-management capacity for the examined variables.

We recommend that hospitals introduce consistent work task descriptions with the necessary elements for effective leadership at the middle organizational level. The employee-development strategy in hospitals should define the knowledge and skills that employees are expected to master, the organizational culture and values, the development of employees at their specific field of expertise, the development of necessary additional knowledge and skills and the minimum requirements in managerial skills for conducting the tasks required by each position.

Furthermore, we recommend that hospitals encourage the accreditation of quality postgraduate programmes in health-care management, comparable to those offered in other European countries (specialist, master’s, and doctoral programmes). Managers and leaders at the second and third leadership levels should be encouraged to obtain any currently lacking knowledge and skills. Specialization programmes for health-care experts should not only include the specific competences required by the different areas of expertise, but also general competences.

The knowledge acquired and enhanced by managers in the proposed education and training programmes constitutes the basis for their work in a modern health-care organization. If we wish to bring about positive changes in organizational culture, the education of middle management must become mandatory. Only individuals who possess the required knowledge should be allowed to fill a certain position, and those already in such positions should be required to gain the necessary knowledge and skills in a specified period. Top managers are responsible for implementing a modern employee-development model.

The hospitals are state owned, which means that the state should develop a strategy on how to improve leadership and management in Slovenian hospitals. There is a need to define the responsibilities of external members of health-care institution councils transparently; these members are appointed by the founder of the institution. Their appointment should not be politically or socially motivated. Instead, a clear goal should be kept in mind: to ensure the most efficient management of funds allocated for meeting the needs of the healthy and ill populations by employing modern leadership and management approaches. Only when each health-care institution is managed efficiently will Slovenia’s health-care system be able to function comprehensively. Here, Slovenia has plenty of opportunities for improvement both in terms of management and financing of the health-care system.

## Abbreviations

MHA: mean health administration; MHC: mean health care; SD: standard deviation.

## Competing interests

The authors declare that they have no competing interests.

## Authors' contributions

BSS prepared the proposal of this article from a broader study entitled “Definition of skills and competences for hospital middle management”. BSS defined the variables and statistical handling. AR participated in the completion of the proposal in the introduction, discussion and conclusion. Both authors read and approved the final manuscript.
